# P-802. Assessing the Benefit of Adding a Rapid Antibiotic Susceptibility Platform for Bloodstream Infections to a Well-Established Stewardship Program within an Academic Health-system

**DOI:** 10.1093/ofid/ofaf695.1011

**Published:** 2026-01-11

**Authors:** Colter G Sheveland, Cassandra Ferguson, Kayla Antosz, Sarah Al Mansi, Caroline Jozefczyk, Andrew B Gainey, Robert Daniels, Joseph Kohn, Chengwen Teng, Anna-Kathryn Burch, Majdi N Al-Hasan, P Brandon Bookstaver

**Affiliations:** Prisma Health – Richland Hospital, Columbia, South Carolina; University of South Carolina College of Pharmacy, Marlton, New Jersey; Prisma Health - Richland Hospital/Antimicrobial Stewardship Collaborative of South Caroline (ASC-SC), Columbia, South Carolina; University of South Carolina School of Medicine - Prisma Health Midlands, Columbia, SC; Prisma Health - Upstate, Greenville, South Carolina; Prisma Health Children's Hospital - Midlands, Columbia, South Carolina; Prisma Health Children's Hospital-Midlands, Columbia, South Carolina; Prisma Health Midlands, Columbia, South Carolina; University of South Carolina School of Pharmacy, Columbia, South Carolina; Prisma Health Midlands/University of South Carolina School of Medicine, Columbia, South Carolina; University of South Carolina School of Medicine, Columbia, South Carolina; Prisma Health Richland - University of South Carolina, Columbia, South Carolina

## Abstract

**Background:**

Rapid diagnostic tests for pathogen identification and antimicrobial susceptibility testing (AST) have enhanced clinicians’ ability to promptly initiate appropriate antimicrobials and streamline regimens. The purpose of this study was to evaluate the benefit of adding the Accelerate Pheno® (AP) system for rapid AST to an existing blood culture identification multiplex polymerase chain reaction (mPCR) workflow in Gram negative bloodstream infections (GNBSIs).

**Methods:**

A multi-centered, retrospective, quasi-experimental study was conducted in patients hospitalized with aerobic GNBSIs between August 2022 and August 2024. Notable exclusions were patients with polymicrobial GNBSIs and those who were no longer hospitalized prior to initiation of antibiotic therapy or blood culture results. The primary endpoint of time to first appropriate streamlined therapy (FaST) was compared between an AP group and post-AP group during a 3-month period. Key secondary endpoints included time to first and final AST, time to de-escalation and notable discordances in AST.

**Results:**

Among 355 charts reviewed, 228 patients were included in the analysis (125 in the AP group and 103 in the post-AP group). Baseline characteristics were similar between groups. A faster median time to initial susceptibilities was observed in the AP-group (39 h vs. 65.5 h, p< 0.0001). However, a median difference of 23.3 h to final susceptibilities was observed between the AP-group and the post-AP group (93.2 h vs. 69.9 h, p=0.008). A total of seven AST discordances were identified in the AP-group, four of which were major errors (false resistance). There was no significant difference in the median time to FaST (22.8 h vs. 11.6 h, p=0.2) or median time to de-escalation (41.9 h vs. 42 h, p =0.527).

**Conclusion:**

The addition of the AP system did not appear to have a meaningful impact on time to FaST or time to de-escalation compared with a historical mPCR and antimicrobial stewardship program (ASP) workflow, despite a more rapid availability of first AST. The study findings may be explained by local susceptibility rates and a well-established ASP that performs prospective audit and feedback on all positive blood cultures with early antibiotic optimization guided by prediction tools and mPCR results.

**Disclosures:**

All Authors: No reported disclosures

